# Frailty and the Interactions between Skeletal Muscle, Bone, and Adipose Tissue-Impact on Cardiovascular Disease and Possible Therapeutic Measures

**DOI:** 10.3390/ijms24054534

**Published:** 2023-02-25

**Authors:** María Elena Soto, Israel Pérez-Torres, María Esther Rubio-Ruiz, Agustina Cano-Martínez, Linaloe Manzano-Pech, Verónica Guarner-Lans

**Affiliations:** 1 Department of Endocrinology, Instituto Nacional de Cardiología Ignacio Chávez, Mexico City 14080, Mexico; 2 Department of Cardiovascular Biomedicine, Instituto Nacional de Cardiología Ignacio Chávez, Mexico City 14080, Mexico; 3 Department of Physiology, Instituto Nacional de Cardiología Ignacio Chávez, Mexico City 14080, Mexico

**Keywords:** frailty, skeletal muscle, bone, adipose tissue, cardiovascular diseases

## Abstract

Frailty is a global health problem that impacts clinical practice. It is complex, having a physical and a cognitive component, and it is the result of many contributing factors. Frail patients have oxidative stress and elevated proinflammatory cytokines. Frailty impairs many systems and results in a reduced physiological reserve and increased vulnerability to stress. It is related to aging and to cardiovascular diseases (CVD). There are few studies on the genetic factors of frailty, but epigenetic clocks determine age and frailty. In contrast, there is genetic overlap of frailty with cardiovascular disease and its risk factors. Frailty is not yet considered a risk factor for CVD. It is accompanied by a loss and/or poor functioning of muscle mass, which depends on fiber protein content, resulting from the balance between protein breakdown and synthesis. Bone fragility is also implied, and there is a crosstalk between adipocytes, myocytes, and bone. The identification and assessment of frailty is difficult, without there being a standard instrument to identify or treat it. Measures to prevent its progression include exercises, as well as supplementing the diet with vitamin D and K, calcium, and testosterone. In conclusion, more research is needed to better understand frailty and to avoid complications in CVD.

## 1. Introduction

Frailty is a global health problem, having an important impact on clinical practice. Its prevalence is increasing, alongside aging, in the population [1,2], increasing from 2.5–3% in adults aged 65 years to 30–35% in those older than 85 years [3]. The clinical syndrome of frailty is complex, having many causes and diverse contributing factors, and it implies an impairment of many systems, resulting in a decrease in the physiological reserve and an elevated vulnerability to stress [1,4].

**Figure 1:**
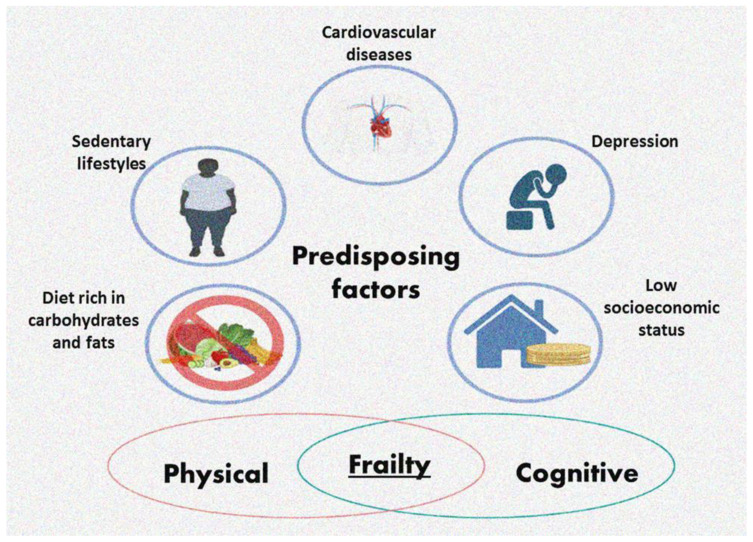
Predisposing factors contributing to the development of frailty.

Frailty is a multidimensional syndrome, rendering its identification and assessment difficult, and there is still no standard instrument to identify it [5]. Subjects with frailty have an elevated risk of mobility limitation, falls, and fractures, as well as an increased length of hospital stays, hospital readmission, and mortality [5]. The risk of developing frailty increases in patients with comorbidities, such as cardiovascular diseases (CVD), a poor diet, a sedentary lifestyle and low socioeconomic position [5] (Figure 1). Mexican–American elderly adults with weight problems are more susceptible to become frail over time [6]. There is also a reciprocal interconnection between depression and frailty in elderly adults [7]. Frailty overlaps with other diseases, including sarcopenia and cachexia (Figure 1) [8].

An analysis of the preventive measures and treatment strategies for frailty is required, since a cure is still not available. It is also important to evaluate new approaches of nutrition, physical activity and medication that could effectively prevent the devastating complications and consequences of frailty, sarcopenia, osteoporosis and cardiovascular damage in the elderly. To avoid frailty, preventive management, including a healthy weight management plan, should be proposed. Moreover, nowadays, psychosocial and nutritional factors are also being recommended [5,9,10,11,12]. In this paper, we discuss the pathophysiology of the syndrome of frailty, including its predisposing factors, and features such as inflammation and oxidative stress. We discuss the interconnections between skeletal muscle, bone and adipose tissue, the association of frailty with CVD and aging, and possible treatments.

## 2. Physical and Cognitive Frailty

Frailty has a physical and a cognitive component [13]. Physical frailty is characterized by diminished muscular strength, endurance, and reduced physiological function, and therefore, it elevates the vulnerability of individuals to become dependent, as well as the risk of death [1]. Physical frailty has been related to disability and sarcopenia [14,15,16]. Physical frailty can be considered to be a pre-disability stage, defined as a need of assistance with basic activities of daily living [15], and it is mainly caused by the loss of muscle quantity and function [17].

Cognitive frailty is defined as a clinical condition in which the presence of cognitive impairment and physical frailty coexist [13,18]. This condition is related to age, and its diagnostic criteria include the presence of mild cognitive and physical impairment without the presence of Alzheimer’s disease (AD), additional dementias, or pre-existing brain disorders [13]. There is also evidence indicating that physical frailty could increase the risk of cognitive impairment, and that cognitive decline may increase the chance of developing physical frailty [19,20]. Accompanying the progressive decline in cognitive function are a loss of appetite and low protein intake, linking cognitive impairment to physical frailty [21].

At least two models to determine frailty have been proposed: the Fried’s frailty phenotype (FP) and the frailty index of the accumulation of deficits (FI). The FP focuses on physical frailty, and takes into account physical performance and muscle strength. According to this model, subjects are classified as robust, pre-frail or frail, depending on the presence of one or more criteria [21]. The use of the FI considers the accumulation of several physical and psychosocial factors in order to identify a frail phenotype, and frailty is mathematically derived and defined as a proportion of the deficits present in an individual. Unlike FP, FI considers different dimensions of frailty: physical capacity, cognition, and quality of life [22]. Additionally, the World Health Organization (WHO) has recently proposed the intrinsic capacity (IC) as a multidimensional indicator of health, including determinations of mental and physical capacities that are that are required by elderly people to continue doing their daily activities. The IC framework includes the cognitive, psychological, sensory, locomotor, and vitality domains. This holistic concept permits a better understanding of different aging pathways, and thereby captures heterogeneity [23]. Additionally, the FRAX fracture risk assessment tool has been successfully used to measure the risk of fractures [24].

**Figure 2:**
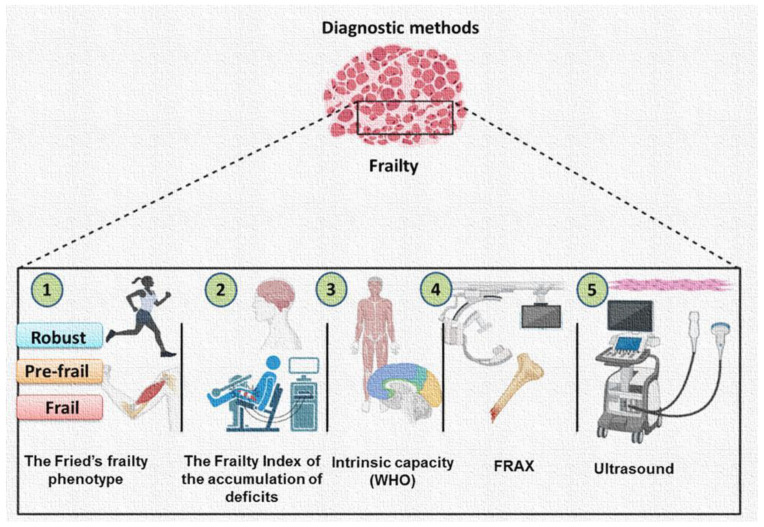
Diagnostic methods. (1) The Fried´s frailty phenotype focuses on physical frailty, and takes into account physical performance and muscle strength. (2) The frailty index considers different dimensions of frailty: physical capacity, cognition, and quality of life. (3) The intrinsic capacity (IC) is a multidimensional indicator of health, including determinations of mental and physical capacities. (4) The FRAX is used to measure the risk of fractures. (5) Ultrasound is a supportive tool for determining sarcopenia and frailty. Abbreviation: WHO = World Health Organization.

Regarding the available tools for the identification and assessment of frailty, there is still no standard instrument to identify it; however, sarcopenia and frailty can be measured by imaging techniques and the use of ultrasound has been proposed to evaluate muscle, in order to support diagnosis and follow-up [25].The application of muscle ultrasounds in sarcopenia and frailty is already under study in very specific conditions [26] (Figure 2).

## 3. Genetic and Epigenetic Bases of Frailty

Aging is associated with frailty, and it is the consequence of genetic and epigenetic factors, nutrient-sensing systems, including the insulin/insulin-like growth factor-1 (IGF-1) signaling pathway, mitochondrial dysfunction, cellular senescence, stem cell exhaustion, inflammation, and some hormonal systems. A comprehensive explanation of aging is provided by the current hallmarks of this stage of life, which include an instability of the genome, decreased length of the telomeres, epigenetic alterations, mitochondrial dysfunction, loss of proteostasis, disturbed regulation of nutrient sensing, cellular senescence, exhaustion of stem cells, modified intercellular communication, compromised autophagy, modifications of the microbiome, altered mechanical properties, dysregulation of splicing, and inflammation, among others [27]. Although the factors involved in aging have been fairly well-defined and genetic causes of aging have been proposed, the factors involved in frailty are primarily inflammation and hormones, with only an anecdotal role for genetic and other potential factors being reported [28]. However, epigenetic clocks, also known as DNA methylation (DNAm), acting as attractive indicators of biological age, have recently been proposed to determine age and frailty. These clocks estimate the biological age of a tissue, cell or organ, based on the levels of DNAm in multiple CpGs across the genome [29,30]. They also suggest that the dysregulation of transcriptional and chromatin networks is a crucial component of aging and frailty [28,31]. The methylation-based mortality risk score (MRscore) and the epigenetic clock-derived age acceleration (AA), which are epigenetic clocks, are independently associated with frailty, and this association is stronger for the MRscore than for the AA. The MR score association only persists with three indicators in regression models [30].

Alterations related to maternal health, premature birth, or congenital alterations can condition the appearance or prevalence of frailty throughout life, acting as epigenetic factors. Maternal dietary and non-dietary factors can influence bone health in offspring. The inclusion of calcium and the levels of 25-hydroxyvitamin D [25 (OH) D] in the diet of the mother, and the effects of maternal supplementation with micronutrients for bone mineral density, are associated with bone mineralization in offspring, particularly in adolescence. Therefore, when the mother’s bone health is optimized, both the incidence of fractures in childhood and frailty in adulthood can be prevented [32,33].

Metabolic bone disease of prematurity results from prenatal and postnatal factors. It manifests as demineralization of the skeleton, which, when aggravated, can cause fragility fractures in the long bones and ribs during daily handling. It is known that 80% of fetal mineral accumulation occurs in the third trimester of pregnancy; in this way, children who are born prematurely lack this mineral contribution at birth. Bone resorption is generally observed, which is difficult to reverse with postnatal treatment. Remineralization occurs with treatment that includes diuretics and corticosteroids; phosphate supplementation; however, tends to worsen this metabolic bone disease of prematurity, because it generates bone loss secondary to hyperparathyroidism [32,33].

The microbiota also acts as an epigenetic factor affecting aging and possibly frailty, since it communicates with the host through various biomolecules and nutrient-signaling-independent pathways. The microbiota becomes more diverse and variable with age. However, when measures of biological age are used with adjustments for chronological age, the overall richness decreases, while certain groups of bacteria associated with frailty increase. Thus, the changes in the microbiota could be used to indicate frailty [34].

In contrast to the scarce evidence of genetic associations with frailty, a genetic overlap of frailty with cardiovascular disease and its risk factors has been reported, focusing on single nucleotide polymorphisms in genome-wide association studies. These studies have shown that frailty is associated with common genetic polymorphisms, many of which are implicated in CVD. Therefore, these studies support the hypothesis of a shared pathophysiology between the entities [35].

## 4. Causes of Frailty: From Increased Protein Degradation or Decreased Synthesis to Inflammation and Oxidative Stress

Frailty is accompanied by a loss and/or poor functioning of muscle mass. Muscle mass depends on fiber protein content, which is a consequence of the balance between protein breakdown and synthesis [36]. Changes in protein intake from the diet and their regulation by anabolic molecules, such as insulin and insulin-like growth factor 1 IGF-1, including their tissue sensitivity, determine protein synthesis in skeletal muscle [37,38,39,40,41,42,43,44]. Protein degradation occurs through autophagy, which is the breakdown of components of the cytosol, including organelles within lysosomes and vacuoles. In this process, multiple protease systems participate, including the ubiquitin–protease system (UPS) pathway, the lysosomal autophagy pathway, and the calpain system [45,46,47,48].

Myostatin is a myokine produced and released by myocytes that inhibits muscle growth by inhibiting the PI3K–Akt–mTOR pathway. By binding to its receptor, it also regulates mitochondrial biogenesis [49]. Mitochondrial activity is essential to maintain muscular energetic homeostasis.

Other neurohormones have been included among the mechanisms that regulate the synthesis and degradation of muscle fibers, since alterations in their concentrations may contribute to imbalances in the anabolic and catabolic pathways [50]. The hormonal signals involved include leptin, ghrelin, melanocortins, neuropeptide Y, and growth hormone [51]. Anabolic agents act by causing impairments in the growth hormone/IGF-1 axis, as well as by increasing insulin resistance. During frailty, there is also an inappropriate hypothalamic response to mechanisms controlling energy homeostasis, such as leptin and ghrelin [52]. Despite the changes in the leptin and ghrelin concentrations, energy intake is not increased, due to a persistent activation of the proopiomelanocortin system, which is anorexigenic. A decreased activity of the neuropeptide Y (NPY, orexigenic) neurons may also contribute to the maintenance of energy intake [40]. Catabolic agents that participate include increased levels of catecholamines and an increased cortisol/dehydroepiandrosterone ratio [51].

**Figure 3:**
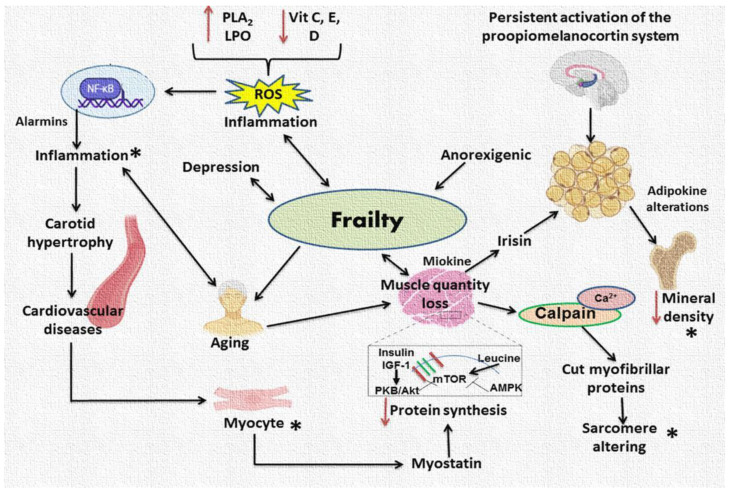
Summary of the different topics reviewed in this article. Development of frailty; inflammation as the main pathophysiological feature and systemic alterations related to it. Abbreviations: Ca^2+^ = Calcium, IGF-1 = Insulin-like growth factor 1, TOR = Mammalian target of rapamycin, LPO = Lipoperoxidation, NF-κB = Nuclear factor kappa b, PLA_2_ = Phospholipase A_2_, ROS = Reactive oxygen species, * = Interconnected routes in frailty.

Alterations in inflammation and oxidative stress also cause frailty. Frail patients have increased levels of oxidative stress biomarkers and proinflammatory cytokines (Figure 3). They also have diminished concentrations of endogenous antioxidants. Inflammation is possibly the main pathophysiologic feature in frailty, having its root in inflammaging [53].

Increased inflammation and a high level of reactive oxygen species (ROS) increase the signaling of protein degradation via numerous key pathways, such as the FoxO transcription factors and nuclear factor-kappa B (NF-κB) [54] (Figure 2, left side). Under normal conditions, there is a balance between the processes of mitochondrial fusion, fission and mitophagy, which regulate the levels of these organelles and favor the function of skeletal muscle. Nevertheless, factors such as inflammation, oxidative stress, and hyperglycemia alter the mitochondrial dynamics [55,56,57,58].

Oxidative stress activates several transcription factors such as NF-κB, AP-1, p53, hypoxia-inducible factor-1 alpha (HIF-1α), peroxisome proliferator-activated receptor gamma (PPAR-γ), β-catenin/Wnt, and erythroid 2-related factor 2 (Nrf2). These transcription factors enhance the expression of more than 500 genes, including those encoding growth factors, inflammatory cytokines, chemokines, cell cycle regulatory molecules, and anti-inflammatory molecules [59]. There are increased oxidative stress levels in aged muscles [60] (Figure 3).

Inflammation and oxidative stress mediators have been proposed as biomarkers for frailty, since they are increased in people with this condition. They are also elevated in subjects with CVD [61]. There is a link between C-reactive protein, IL-6, fibrinogen, and white blood cell count and frailty [61]. An elevation in alarmins and inflammatory cytokines in peripheral blood is also related to poor cardiovascular outcomes in frail patients [62].

Markers of oxidative stress, including lipoprotein phospholipase A_2_, isoprostanes, malonaldehyde, 8-hydroxy-20-deoxyguanosine, derivate of reactive oxygen metabolites, and protein carbonylation levels, have been associated with frailty in small cross-sectional studies [63]. Limited evidence of lower antioxidant parameters, such as vitamin C, E, and α-tocopherol, in frail patients has also been reported [63]. It is uncertain whether these associations are causal.

The participation of inflammation and oxidative stress in the frailty process has also been tested in experimental models [64,65,66,67,68,69,70,71].

## 5. Interactions between Bone, Skeletal Muscle, and Adipose Tissue

Frailty is associated with osteosarcopenia, a condition whose prevalence is unclear. Osteosarcopenia was present in patients during the recent COVID-19 pandemic, revealing the importance of the initiation of preventive care in the treatment of alterations in bone metabolism, prior to a state of frailty [72,73]. Therefore, interconnections between bone and skeletal muscle are important to assess. Bone is the main structural connective tissue, and it is continuously undergoing regeneration by osteoblasts and absorption by osteoclasts. Osteoporosis is a disease characterized by low mass and the micro architectural deterioration of bone tissue, due to an increased activity of osteoclasts, as well as a decrease in osteoblast function and calcium absorption. Chronic low bone mineral density can adversely impact the mobility of the patients, their function, and quality of life. Homeostasis of bone resorption/formation is regulated by factors derived from several tissues, including adipose tissue and skeletal muscle. The relationship between fat and bone is complex and controversial. Adipose tissue is an endocrine organ that is closely associated with bone mass density. Several studies have shown that adipocytes such as resistin, leptin, adipsin, and adiponectin have effects on bone differentiation and metabolism [74]. Particularly, leptin was reported to suppress bone genesis and to increase bone erosion via the receptor activator of nuclear factor (NF)-κB ligand (RANKL). It also induces osteoblast activity and mineralization and affects bone through its actions on the hypothalamus [75] (Figure 3). On the other hand, the beneficial effects of adiponectin on increased bone formation and decreased resorption are controversial, and depend on age, gender, estrogen levels, bone sites, and the levels of proinflammatory cytokines [76,77,78]. Regarding this aspect, chronic inflammatory diseases promote bone loss due to factors such as IL-6 and TNF-α, and they suppress osteoblast differentiation and enhance osteoclast differentiation [76]. In addition, there is a strong relation between fat tissue and the loss of muscle mass, leading to sarcopenic obesity, which is defined as a gradual loss of skeletal muscle mass and strength in patients with excess fat [77]. In health conditions, adipose tissue releases leptin that stimulates glucose uptake and the growth of skeletal muscle, through the activation of AMP-activated protein kinase (AMPK) [78]. Moreover, obesity induces an increase in the release of pro-inflammatory cytokines, and resistance to leptin is present; hence, sarcopenia occurs. Locally, oxidative stress, mitochondrial dysfunction and insulin resistance contribute to the development of sarcopenic obesity in skeletal muscle [77].

Currently it is known that bone can produce several substances, including proteins, growth factors and cytokines, that regulate bone mass and metabolism. Osteocalcin is an abundant, non-collagenous, vitamin K-dependent protein synthesized by osteoblasts. Approximately 10–30% is released into the circulation, having effects on other peripheral tissues. Osteocalcin may increase insulin sensitivity through binding to a G-protein-coupled receptor called GPR6A, and it is related with fat mass; however, the mechanism regarding the effect of osteocalcin in human metabolism is still unclear [79,80]. Osteoprotegerin is a glucoprotein synthetized in bone that belongs to the TNF receptor family, and it is associated with obesity, Type 2 diabetes, inflammation, and vascular calcification. Several studies suggest the protective role of osteoprotegerin against osteoporosis; however, its effect on bone mass is still unclear [78,79] (Figure 3).

Calcium metabolism and the change in serum parathyroid hormone levels are involved in bone homeostasis and in the lipogenesis carried out by adipocytes. Furthermore, they participate in the mechanisms that lead to increased systolic blood pressure, the development of atherosclerotic plaques, and cardiovascular events [80,81,82,83,84,85,86,87].

Including the assessment of calcium metabolic status, coronary calcium, and serum low density lipoprotein (LDL) levels in the cardiovascular risk profile of patients, and improving bone mineral density could contribute to the prevention of CVD.

Bone and muscle are closely interrelated, leading to a complex network called the musculoskeletal system. It has even been proposed that the loss of skeletal muscle mass and function (sarcopenia), affects bone mass, and increases the risk of falling and fragility fractures [88]. Muscle can synthesize and secrete a series of myokines, which play important roles in the growth, development, and function of the bone [89]. Irisin is a myokine associated with thermogenesis and energy expenditure in adipose tissue, and it stimulates osteoblast differentiation, proliferation, and mineralization. Its levels increase with physical activity [73,88,90]. On the other hand, myostatin has a detrimental effect on bone remodeling by activating the RANKL pathway [90]. Moreover, several studies have suggested that a loss of skeletal muscle mass may contribute to metabolic disease [91] (Figure 3).

Loss of body mass and osteoporosis often occur during aging. Bone and muscle constitute endocrine targets and they also act as endocrine organs, sending and receiving paracrine and endocrine signals. Bone mineral content correlates with muscle mass during growth. Osteoporosis and the loss of muscle mass share pathophysiological factors in men and women [92]. Frailty and osteoporosis are age-related and feature, as an underlying basis, chronic inflammation, changes in body composition, and hormonal imbalances [93,94].

## 6. Frailty and Aging 

There is an increased prevalence of frailty in the elderly population, and aging is related to the loss of muscle mass and strength, which may be prevented by exercise. A lack of exercise with the consequent loss of skeletal muscle mass is now considered a frequently overlooked, non-traditional cardiovascular disease risk factor. Exercise has antiatherogenic effects on blood vessels, balances the autonomic input to the body, protects against injury by ischemia–reperfusion, has anti-inflammatory effects, and stimulates myocardial regeneration [93].

Although frailty may occur during various conditions that are independent of age, aging is an important factor predisposing one to its development. Aging is multifactorial, including a combination of physiological changes, such as a decline in smell and taste, a reduced desire to eat, delayed gastric emptying, and pathological conditions, among which depression, dementia, somatic diseases, medications and iatrogenic interventions, oral health status, dehydration, and social factors such as poverty and loneliness are included [94]. Loneliness, poverty, and social isolation are the predominant social factors that contribute to decreased food intake in the elderly population. These factors may result in chronic depression, which is a common psychological problem in the elderly and a significant cause of loss of appetite with the consequent loss of muscle mass [95].

A loss of sex hormones in men (andropause) and in women (menopause) is also associated with aging. Andropause in men may lead to a reduction in bone mass and density and in a decrease in muscle strength. Menopause, and the decline of 17β-estradiol in women, results in the severe decalcification of bone, arthritis, and promotes the activation of the inflammatory cytokines, hip fracture, presence of pressure ulcers, and a decrease in the immune function [96]. Cachectic males have greater deficits in handgrip strength when compared to cachectic females [97].

## 7. Frailty and Cardiovascular Diseases

Elderly people not only lose skeletal muscle, but they are also more susceptible to CVD [1,61,98]. CVDs linked to frailty include heart failure, myocardial infarction, atrial fibrillation, heart valve disease, and stroke [99,100].

Risk factors for CVD that are accompanied by inflammation and oxidative stress appear to be shared with frailty, including a lack of physical activity, smoking, an inappropriate diet and obesity, particularly abdominal fat accumulation, hypertension, and diabetes [53,98]. Moreover, other potentially modifiable risk factors for frailty are preclinical atherosclerosis, inflammation, and decreased bone mineral density of the femoral neck. There is a strong connection between aging, increased common carotid artery intima–media thickness, increased inflammation, and decreased bone mineral density of the femoral neck [99,100].

Frailty constitutes an unfavorable prognostic factor in patients with CVD, and, in turn, CVD increases the risk of frailty [98]. In fact, frailty could be considered, in the future, as a risk factor for CVD. Moreover, the progression to severity of patients with CVD is common in individuals with frailty [101]. Frail patients with heart failure are often predisposed to poor outcomes and, therefore frailty is a relevant prognostic factor.

### 7.1. Frailty and Heart Failure

Frailty and heart failure share common antecedents and are associated with each other. Therefore, it is necessary to develop more comprehensive evaluation techniques, since the existing ones have not been validated. Better therapeutic interventions for frailty to improve the prognosis and quality of life of frail patients with heart failure and another CVD are required [102]. Emphasis on the measurement of frailty in patients with cardiovascular disease, particularly in patients with HF and in patients who require cardiac surgery for any etiology, and regardless of age, should be promoted, since detecting frailty could improve patients’ outcomes. Assessing only the physical domains of frailty, such as involuntary weight loss, exhaustion, muscle weakness, slow walking, and low levels of physical activity, independently of age is not enough, since there is a relationship between physical frailty and CVD that may be bidirectional [103]. Other non-physical frailty domains, such as cognitive, psychological, and social components, should also be considered [104].

Although there is growing interest in evaluating frailty, no validated instrument to measure frailty in the population with heart failure was found in a meta-analysis, rendering monitoring and the comparison between different populations more difficult [105].

In patients with cardiovascular damage that require surgery or interventional management, the outcomes may be difficult, and they may face prolonged hospital stays in the cases of previous nutritional and mobility disorders. The evaluation of frailty still requires validation in various populations. More research is needed to have a better understanding of frailty when it is present together with comorbidities and cardiovascular damage, in order to avoid complications.

### 7.2. Frailty, Blood Pressure, and Heart Rate

Alterations in blood pressure can also have an impact on the appearance and development of frailty. In fact, orthostatic hypotension is associated with syncope, falls, and fragility fractures. Under hypotensive conditions, the management of cardiovascular risk factors and cardiovascular disease is difficult, and can become complicated leading even to terminal stages [106]. There is also susceptibility to vascular fragility that is related to primary pulmonary hypertension [107].

On the other hand, vascular damage due to hypertension has an important correlation with alterations of the nervous system, and, consequently, brain frailty. Age-associated hypertension can trigger microvascular damage in the brain and kidney. In the renal, vertebral, and carotid arteries, branching into small arterial vessels, high pressure fluctuations occur due to low resistance. These fluctuations increase by three to four times with age. The highly pulsatile flow faced by vessels subjected to high pressures is associated with microvascular damage, intellectual impairment, and renal failure [107,108].

Blood pressure variability and heart rate variability may possibly be markers for frailty, because they help clinicians understand changes in cardiac autonomic modulation. Altered endothelial function increases the production of ROS and inflammation, and underlies alterations in the variability of blood pressure [108,109,110].

## 8. Treatment of Frailty

Preventing and reversing frailty requires an integrative approach, in which physical activity, nutritional interventions, cognitive training, and possibly a pharmacological approach may be included. Although evidence is still weak and more research is needed, a combined intervention with exercise and nutrition as a basis, and pharmacological treatment as a supplement, could help these patients [111]. The possible treatments for frailty are discussed in the next sections and are summarized in Table 1.

### 8.1. Non-Pharmacological Strategies; Exercise and Nutrition

Physical exercise, as a non- pharmacological strategy, is highly effective in reducing functional decline and frailty. The role of this kind of non-pharmacological strategy is highly underestimated in elderly adults [111]. Physical exercise reduces the relative risk of developing functional limitations among elderly subjects reporting regular- and moderate-intensity physical activity. Low levels of physical activity are a major risk factor for both frailty and CVD. Muscle strength and endurance are both strongly influenced by usual levels of physical activity, and can be modified by increasing exercise [112]. In this sense, muscle weakness hinders dynamic weight-bearing exercise and has a negative impact on bone development.

Disuse of the skeleton due to prolonged lack of exercise also delays bone growth [113]. However, exercise, such as walking and weight-lifting, stimulates bone regeneration and dynamic-loading activity, leading to greater gains in bone tissue. In addition, resistance training several times per week promotes an adequate access to energy, and protein intake is necessary for it to be effective [110,112].

Physical activity also preserves, and possibly improves, cognitive function in people without dementia, reducing cognitive decline by around thirty percent [114]. On the other hand, cognitive training is used as an approach toward capitalizing on neural plasticity, by targeting and training cognitive domains. Therefore, cognitive training has emerged as an efficient behavioral strategy to improve or maintain cognitive health in old age [114].

Malnutrition plays a key role in the pathogenesis of both frailty and sarcopenia; nevertheless, strong evidence to base recommendations on is still unavailable. Protein, leucine, omega-3 fatty acids, and vitamin D (vit D) play a significant role in the prevention and treatment of sarcopenia and functional decline [110]. Essential amino acid supplements, including leucine, showed some effects in improving muscle mass and functional parameters. Protein supplements did not show consistent benefits in terms of muscle mass and function. Consuming a Mediterranean-style diet is associated with a significantly lower risk of frailty [114].

The evaluation of frailty is fundamental for the prognostic evaluation of the patients to whom cardiac rehabilitation therapy will be applied, and frailty constitutes a fundamental therapeutic approach in perioperative management [115].

### 8.2. Vitamin D

The degree of progression of muscular frailty and the development of osteoporosis are strongly linked to low levels of vit D, and they is also associated with a decrease of its receptor, VDR [116]. Most of vit D is synthesized in the skin (70–80%), during exposure to ultraviolet rays, and 20% is incorporated via food ingestion of sardines, fish liver oils, liver from cattle, egg yolks, mushrooms, cheese, and milk. Exposure of the skin to the sun for 15–20 min/day induces the conversion of 7-dehydrocholesterol to pro-vit D, which is synthesized from cholesterol. This compound is then transported to the liver, where it is hydroxylated to 25(OH)-vit-D and then released again to the circulation, reaching the kidney. In the kidney, it is again hydroxylated to 1,25 dihydroxy vit D, denominated calcitriol, which is the active form [1].

Calcitriol enters the cells by diffusion, crossing the membrane because of its lipidic nature, and it then joins to the nuclear receptor and transcription factor VDR [117]. This receptor mediates both the genomic and non-genomic effects of vit D, such as the regulation of calcium and phosphate metabolism, and bone mineralization [118]. The human VDR gene is located in the q arm of chromosome 12 and consists of nine exons, encoding 427 amino acids [119]. However, age, sex, and pathological conditions modulate the expression of VDR in muscle tissue. The deficiency of VDR may lead to hypertrophy, as well as changes in strength, weakness and lower mass of the muscle fibers, which are associated with frailty. Deficiencies in vit D result in decreased type II fiber diameter and area, and treatment with 1-α-hydroxy vit D3 and calcium restores this condition [1].

In obese patients, particularly those treated with corticosteroids, higher doses of vit D of up to 1000 IU/day are needed because of its low bioavailability [120]. Moreover, the use of the hydroxylated vit D3 metabolite calcifediol, which does not require hepatic activation by the CYP system, and which is also involved in the native hydroxylation of vit D, is recommended. Therefore, the monitoring of serum 25-OH-vit D levels in patients with frailty would be strongly required. The adjustment of the supplementation of vit D according to the muscular mass index is also recommended if the concentration levels of 25-OH-vit D are low, considering the detrimental effects of vit D deficiency on muscle performance [121].

In different pathologies associated with muscular damage, such as sarcopenia, cachexia, and muscular frailty, a combined treatment, including vit D and anti-osteoporotic drugs, such as bisphosphonates (BPs), calcium supplements, and androgens [122], is recommended.

### 8.3. Calcium

Calcium supplements have shown short-term improvements in terms of bone mineral density. Moreover, intestinal calcium absorption is dependent on other factors, such as vit D levels. Independently of whether the homeostasis of circulating vit D levels can be achieved through supplementation in patients with frailty, a calcium-rich diet based on dairy products is strongly suggested [123]. Therefore, a balanced calcium-rich diet is recommended as the best long-term strategy to maintain adequate calcium levels in patients with muscular frailty [124].

### 8.4. Vitamin K

This fat-soluble molecule is mainly present in green, leafy vegetables, and it is produced by the gut microbiota, acting as a cofactor on bone proteins such as osteocalcin. This vitamin decreases the activity of NF-κB, it has antioxidant activity, and it improves bone mineralization [125,126]. Vitamin K regulates metabolism and increases cell differentiation in skeletal muscle [127]. Despite the association of insufficiency between vitamin K and frailty, the role of this vitamin on osteoporosis and sarcopenia has not been fully elucidated, and its effects depend on factors including the dose, bioavailability, catabolism, or even genetic elements [127,128,129].

### 8.5. Natural Compounds

Some nutraceuticals and functional foods have beneficial effects on sarcopenia, and could also help in treating frailty, including curcumin, resveratrol, catechin, soy protein, and ginseng, without having significant side effects [130]. In addition, natural compounds that promote osteoblast proliferation and differentiation include flavonoids, glycosides, coumarins, terpenoids, phenolic acids, phenols, and other molecules, such as tetrameric stilbene, anthraquinones, and diarylheptanoids. The bone formation effect induced by natural products is regulated by the expression of bone-specific matrix proteins, transcription factors, signaling pathways (MAPK, BMP), local factors (ROS, NO), the OPG/RANKL system of osteoblasts, and estrogen-like biological activities [131]. Nevertheless, the clinical benefits of plant-derived natural products remain inconclusive, due to limited human trials.

### 8.6. Hormonal Treatments

Regarding treatment using hormones, testosterone supplementation in elderly people elevates fat-free muscle mass, but not strength. Although dehydroepiandrosterone has been proposed as a possible treatment, studies did not show a positive effect for building muscle mass or losing fat mass. Growth hormone supplements increases muscle mass and strength in healthy men over 60 years old, but do not restore the rate of myofibrillar protein synthesis. Additionally, there are frequent side effects of growth hormone replacement. There may also be a role for IGF-1 in frail elderly adults; however, only a small pilot trial has been done, and further assessment of its therapeutic potential in this population is needed. Treatment using angiotensin-converting enzyme inhibitors also seems to slow the decline in muscle strength in elderly women with hypertension and without heart failure [114]. Recombinant human chorionic gonadotropin has also been proposed, with no conclusive results on its use.

Regarding the participation of hormones in cognitive frailty, cortisol plays an important role in the development of depression, which is a complex, multifactorial, and heterogeneous disease, with a high prevalence worldwide. The levels of this hormone are increased during aging. This hormone impacts cognitive capacity and attention [132]. An association between thyroid hormones and frailty has also been found [133].

### 8.7. Pharmacological Treatments

Individual drugs that have shown to improve physical performance, muscle strength, or body composition include alfacalcidol, teriparatide, piroxicam, capromorelin, and empagliflozin, the SGLT2 (sodium glucose cotransporter 2) inhibitor [111,134]. Empagliflozin reduces frailty in diabetic and hypertensive patients, most likely by decreasing the generation of mitochondrial ROS in endothelial cells [135].

Bisphosphonates are primary agents in the current pharmacological arsenal against osteoclast-mediated bone loss due to osteoporosis [136]. Bisphosphonates inhibit bone resorption, bone turnover, and, therefore, bone loss. Their effect is due to a decrease in the generation of new bone remodeling units, and a decrease in the depth of the erosion cavities [137,138,139].

Combinations of drugs for the treatment of the physical and cognitive components of frailty (muscle loss and osteoporosis) are often simultaneously prescribed, due to the multi-faceted nature of frailty, leading to polypharmacy, which may result in potentially inappropriate medications. Preventing polypharmacy has also been suggested to improve frailty, or some of its aspects (Figure 3 summarizes the different topics that were reviewed in this article) [133,134].

**Table 1:** Summary of the possible treatments to combat frailty.

Non-Pharmacological Strategies	Recommendations	Beneficial Effects
Exercise	Walking weight-lifting Resistance training three times per week	Increases muscle mass, bone regeneration, and improves cognitive function. Reduces cardiovascular events and bone fractures [120,121,122,123].
Nutrition	Leucine amino, acid, omega-3 fatty acids and Mediterranean style diet	Improved the muscle mass [110,114,115].
Vitamin D	Exposure of the skin to the sun for 15–20 min/day, and ingestion through sardines, fish liver oils, liver from cattle, egg yolks, mushrooms, cheese, and milk is recommended in obese patients, up to 1000 IU/day	Increases and favors the metabolism of calcium and phosphate, as well as bone mineralization. Increases type II fiber diameter and area [1,116,117,118,119,120,121].
Vitamin K	Vegetable-rich diet	Improves bone mineralization. In skeletal muscle it increases cell differentiation [125,126,127].
Calcium	Balanced calcium rich diet and 1300 mg/day in patients aged 9–18 years, and 1000 mg day between 19 and 50 years	Increases bone mineralization [122,123,124].
Natural compounds	Curcumin-, resveratrol-, catechin-, soy protein-, and ginseng-rich diet.	Osteoblast proliferation and differentiation [130]
	Flavonoids, glycosides, coumarins, terpenoids, phenolic acids, and phenols	New bone formation through bone-specific matrix proteins and transcription factors such as MAPK, BMP, OPG/RANKL system [131].
Hormonal treatments Testosterone and dehydroepiandrosterone Growth hormone Chorionic gonadotropin	Monthly testosterone injections for 2 years	Elevates fat-free muscle mass, Increases muscle mass and strength [114]. Without results.
Pharmacological treatments	Alfa calcidol, teriparastide, piroxicam, capromorelin empagliflozin, sodium glucose co-transporter 2 inhibitor [111,134]	Improvement of physical performance, muscle strength, and body composition.
Bisphosphonates	dose of 1 mg/kg to 1.5 mg/kg in young patients	Inhibits bone desorption, bone turnover, and increase bone mass. It reconstitutes erosion cavities [133,134,136,137,138,139,140,141,142,143].

Abbreviations: MAPK = Mitogen-activated protein kinases, BMP = Bone morphogenetic protein, OPG = The discovery of osteoprotegerin RANKL = The receptor activator of nuclear factor-κβ ligand.

## 9. Treatment of Cardiovascular Diseases in Patients with Frailty

Few studies have focused on the treatment of CVD in patients with frailty. Studies include a variety of aims and designs. Observations are still not conclusive. A summary of the results is shown in Table 2.

## 10. Summary and Conclusions

Frailty is an international health problem that renders clinical practice difficult. It is a complex syndrome, having physical and cognitive components and it is a consequence of many contributing factors, resulting in alterations of many systems, and leading to a reduced physiological reserve and an elevated vulnerability to stress. Frailty is associated with aging and CVD. Genetic causes have been proposed for aging; however, genetic factors in frailty are poorly reported. In contrast, epigenetic clocks have recently been proposed to determine age and frailty. Nevertheless, there is a genetic overlap of frailty with CVD and its risk factors. Frailty is accompanied by a loss and/or poor functioning of muscle mass, and thus depends on fiber protein content. Muscle protein content results from the balance between protein breakdown and synthesis. Inflammation and oxidative stress play an important role in the pathophysiology of frailty and frail patients have increased levels of oxidative stress biomarkers and proinflammatory cytokines. The identification and assessment of frailty is difficult, since this syndrome is multidimensional, and, furthermore, there is no treatment for it. However, several measures can be taken to prevent its progression, such as executing weight- lifting exercises, as well as supplementing the diet with vitamin D, calcium, and testosterone. In conclusion, more research is needed in order to have a better understanding of frailty and to avoid cardiovascular complications.

## Figures and Tables

**Table 2:** Summary of studies on the treatment of frailty and analysis of perspectives in cardiovascular disorders in patients with frailty.

Objective	Design	Observation
To explore specific pharmacotherapy for heart failure in frail hospitalized patients with heart failure.	Transversal Multicentric	Frail patients are less likely to receive HF-specific pharmacotherapy than their non-frail counterparts. There is an association between the use of HF-specific pharmacotherapy and better clinical primary outcomes: days alive and out of hospital DAOH and reduction of mortality at 30 and 180 days in frail patients [144].
To assess differences in functional capacity, frailty, and quality of life at 3 months compared to baseline. To explore the mean change from baseline in 3 months in cardiac remodeling by echocardiography. To measure serum brain natriuretic peptide levels and the incidence of adverse drug reactions.	Randomized Controlled Trial an open-label parallel design,	Testosterone can be detrimental to heart health, due to its ability to prevent myocardial tissue from proliferating and retaining fluid and salt with long-term damage. However, the serum testosterone level may predict the risk of cardiovascular events, with patients with the greatest increase in serum free testosterone level showing a significantly higher risk of such events. However, there are differences in functional capacity, frailty, and quality of life at 3 months compared to baseline. Secondary endpoints included mean change from baseline at 3 months in cardiac remodeling by echocardiography, serum brain natriuretic peptide levels, and the incidence of adverse drug reactions. Logistic regression analysis of this study showed that this effect did not cause significant harm. On the other hand, low testosterone levels can constitute a marker of disease. More studies are required in this context [145].
To investigate the impact of frailty and malnutrition on cardiac prognosis by combining the Clinical Frailty Scale (CFS) and the Geriatric Nutritional Risk Index (GNRI) in patients who underwent percutaneous coronary intervention (PCI).	Prospective	The cardiac prognosis of patients with frailty and malnutrition was poorly investigated by combining the Clinical Frailty Scale and the Geriatric Nutritional Risk Index in patients undergoing percutaneous coronary intervention. This study found that patients with frailty and malnutrition had a higher risk of MACE after percutaneous coronary intervention than patients with only frailty or malnutrition. Post-percutaneous coronary intervention patients should be evaluated if they suffer from of combined frailty and malnutrition. This work suggests a perspective to evaluate and treat patients with frailty while monitoring their heart disease, and if they should undergo PCI [146].
To investigate the effects of dehydroepiandrosterone on cardiovascular risk factors in older women with frailty characteristics	Double-blind, randomized, placebo-controlled trial	There were no consistent effects of dehydroepiandrosterone on cardiovascular risk; however, this study shows that short-term dehydroepiandrosterone therapy is safe for older women with regard to cardiovascular risk factors. This study is novel and recruited women with evidence of physical frailty [147].
To explore the views and experiences of frailty from the perspective of primary care professionals, including nurses, who work directly with elderly people within the community.	Epidemiology	This is an area of opportunity to enhance the early assessment of frailty and to be able to plan preventive multifactorial interventions through effective detection strategies by primary care professionals [148].
To summarize the available evidence on possible cardiovascular risk harms and benefits of vitamin D.	Systematic review	A total of 6509 patients were identified from 13 published journal articles that underwent selection analysis. The role of vitamin D supplementation in CHF was evaluated. CHF patients with sufficient vitamin D had a lower risk of death from all causes, even after accounting for potential confounding variables. There is a need for long-term, fully-enrolled, randomized, placebo-controlled studies of high-dose vitamin D3 supplementation in patients with CHF, caused by left ventricular systolic dysfunction. The possibility of long-term advantages of vitamin D administration is suggested [149].
To examine rehabilitation modalities for patients with heart failure and frailty who require comprehensive intervention.	Review	Frailty comprises multiple domains, where the interventions include exercise, nutrition, and medication; the psychophysiological, cognitive and social state must be evaluated, stratifying these deficiencies. These types of interventions are more necessary than individual intervention in the various symptoms associated with the combination of heart failure and frailty. There is a relationship between frailty and HF prognosis; however, interventions that improve prognosis are not yet well defined. Interventions aimed at improving the prognosis of multidomain frailty are needed [150].
To examine rehabilitation modalities for patients with heart failure and frailty who require comprehensive intervention.	Review	It is relevant to integrate frailty and heart failure through the early detection of both in the outpatient and hospital phase. Classifying and staging the risk of frailty can lead to new management strategies with better results in this vulnerable population [151].
To investigate whether frailty predicts an increased risk of fatal and nonfatal cardiovascular disease incidents among elderly adults living in a community	Cohort	A population cohort of 5015 participants aged 55 years and above, free of CVD at baseline, was followed up for 10 years. Frailty was associated with an increased risk of CVD morbidity, and especially mortality, mediated in part by traditional cardio metabolic and vascular risk factors, comorbid depression and associated cognitive impairment, and chronic inflammation. Given that pre-frailty and frailty are reversible through lifestyle interventions and reduction of the burden of cardiovascular disease and mortality, therapeutic interventions should target the population with pre-frailty and early frailty [152].
To investigate the efficacy of sacubitril/valsartan according to frailty status in 4796 patients with heart failure with preserved ejection fraction.	Randomized clinical trial	Frailty is an increasingly common problem, and frail patients are less likely to receive new pharmacological therapies because the risk–benefit profile is perceived to be less favorable than in non-frail patients. This study investigated the efficacy of sacubitril/valsartan based on frailty status [153].
To investigate the efficacy and tolerability of dapagliflozin according to frailty status in patients with heart failure with preserved or mildly reduced ejection fraction.	Randomized clinical trial	They found that the benefit of dapagliflozin was consistent across the frailty range studied. The improvement in health-related quality of life with dapagliflozin occurred early, and was greater in patients with a higher degree of frailty [154].
To explore the role of frailty, malnutrition, and sarcopenia	Review	Malnutrition is one of the hallmarks of frailty in elderly patients and a predictor of worse outcomes in elderly patients with severe aortic valve stenosis. In this study, the authors comment that it is clear that there are some components that can be intervened on, before or after TAVI, to possibly optimize the results in this population. The process aims to improve the physical and nutritional condition in order to improve the results after TAVI; a response was expected in the study proposed by PERFORM-TAVR (Protein and exercise to reverse frailty in older men and women undergoing aortic valve replacement). A trans catheter [NCT03522454]) is undergoing a randomized, controlled trial that could provide information on whether this intervention could improve TAVI outcomes in elderly patients [155].
To address the role of frailty and sexual differences in the management and prognosis of elderly outpatients with heart failure.	Prospective multicentric	Frailty is common among patients with HF. This study suggests taking the results obtained by some previous studies with caution, since the sample of patients included showed that they had high heterogeneity, and there is evidence that they included patients undergoing an acute episode of HF, in whom frailty was not uniformly evaluated. It has been recommended to evaluate frailty on an outpatient basis, and not in the setting of an acute HF event. In the results of this study, they comment that although the prevalence of frailty was lower, a greater proinflammatory state could explain, at least in part, why there was a greater prognostic impact in older men with HF [156].
To present selected and commonly used tools in the evaluation of shortening fraction in patients with CVD, including those of greatest relevance in cardiac surgery.	Review	The identification of frailty syndrome in cardiac surgery can be performed for different purposes. Screening scales are appropriate for perioperative risk stratification, and these tools may be necessary to define specific and individualized preoperative management strategies, optimize the patient’s condition, and reduce complications. In clinical settings, tools to assess frailty must have the ability to differentiate between potentially reversible and irreversible frailty, so that one objective could be to improve the identification of patients who are candidates for cardiac surgery, and those who survive, to improve their survival and quality of life [157].
To determine the potential causality in association of the frailty index with cardiovascular outcomes in coronary artery disease, myocardial infarction, atrial fibrillation, and heart failure.	Review meta-analysis	They included 184,305 cases and 60,801 controls and reported that there is genetic evidence of a causal association between the frailty index and the risk of CVD. More studies are needed to demonstrate the causality related to the risk of atrial fibrillation [158].
To determine the impact of age and frailty on the pooled outcomes of death, stroke, and myocardial infarction (MI) at 30 days, in patients receiving Carotid endarterectomy or carotid artery stenting, for severe symptomatic carotid stenosis.	Retrospective	Age and frailty have a significant impact on the risk of death, stroke, and myocardial infarction at 30 days in patients with severe symptomatic carotid stenosis who receive carotid endarterectomy, but not carotid artery stenting [159].
To assess the prognostic impact of the coexistence of hepatorenal dysfunction and frailty in patients with heart failure.	Retrospective	Complex multiorgan interactions, such as the coexistence of hepatorenal dysfunction in HF, negatively affect the patient’s prognosis. However, the association between hepatic/renal dysfunction and frailty, and the effects of their coexistence on HF prognosis, remains unclear [160].
To determine the association between frailty and outcomes after TAVI.	Systematic Review 54 studies.	In TAVI patients, frailty is associated with poorer early and late health. The tools that identify frailty have not been evaluated during the selection of patients with aortic stenosis who undergo trans catheter aortic valve implantation (TAVI), therefore, randomized trials are necessary to determine if frailty influences the outcome, or improves or worsens after the intervention, and how treatment should be considered [161].
To analyze frailty as a cardiovascular risk factor (and vice versa).	Meta-analysis	An important association between frailty and CVD is suggested. There is currently more research regarding frailty as a potential risk factor for CVD than that regarding CVD being a risk factor for frailty. It is required to investigate, in both directions, the influence that one has on the other [162].
To discuss the pathophysiology and molecular mechanisms underlying muscle changes during aging, highlighting the role of oxidative stress. To review strategies used to prevent and treat age-associated muscle disorders. To evaluate the role of physical exercise and introduce new agents under development.	Review	CVDs, such as HF, myocardial infarction, atrial fibrillation, severe heart valve disease, and stroke, increase the risk of frailty. CVD comorbidities and frailty are influenced by the same risk factors, including low physical activity, smoking, dietary patterns, obesity, and diabetes. Additionally, many biomarkers, reflecting multisystem dysfunction, are common [60].

## Data Availability

Not applicable.
